# Phéochromocytome et grossesse - Gestion péri opératoire et conduite à tenir obstétricale: a propos d’un cas clinique et revue de littérature

**Published:** 2010-06-11

**Authors:** Hicham Sbai, Yassin Essatarra, Abdelkrim Shimi, Amal Ankouz, Bachir Benjelloun, Abdelmalek Oussaden, Khalid Ait Taleb, Nabil Kanjaa

**Affiliations:** 1 Service d’anesthésie-réanimation A4, CHU Hassan II, Fès, Maroc; 2 Service de chirurgie viscérale A, CHU Hassan II, Fès, Maroc

**Keywords:** Phéochromocytome, Grossesse, Anesthésie, Pronostic

## Abstract

**Résumé:**

L’association phéochromocytome et grossesse est rare pouvant mettre en jeu le pronostic vital maternel et fœtal. Le diagnostic est aisé à condition d’y penser systématiquement face à une hypertension artérielle gravidique atypique, accompagnée de signes cliniques évocateurs, ou résistante au traitement. La certitude diagnostique est donnée par des tests biologiques simples et fiables. Nous rapportons le cas d’un phéochromocytome survenu au 1er trimestre, révélé par des épisodes d’hypertension artérielle. Le traitement avait consisté en une préparation médicale préopératoire suivie d’une surrénalectomie. L’évolution materno-fœtale était favorable. La survenue d’un phéochromocytome au cours de la grossesse pose un problème de diagnostic et de contrôle tensionnel. La stratégie thérapeutique dépend du terme, du retentissement materno-fœtal et de la réponse au traitement médical.

## Introduction

Le phéochromocytome est une maladie rare qui peut être révélée par la grossesse et dont la prévalence est de l’ordre de 1 pour 50 000 grossesses [1]. La démarche diagnostique et thérapeutique actuellement mieux codifiée conditionne le pronostic maternel et fœtal sombre lorsque le diagnostic est méconnu. Le diagnostic repose sur la clinique (signes d’appel), la biologie (diagnostic positif) et la radiologie (diagnostic topographique).

## Patient et observation

Madame S.R âgée de 28ans, primigeste sans antécédents médicaux a été hospitalisée à 12 semaines d’aménorrhée (SA) pour hypertension artérielle (HTA) à 180 /110 mmHg associée à des céphalées, palpitations et bouffées de chaleur, sans oedème ni protéinurie. Sur le plan obstétrical, l’examen clinique, l’échographie et l’étude doppler n’ont pas montré d’anomalie utérine ou fœtale.

L’examen cardio-vasculaire, en dehors d’une tachycardie sinusale, a été sans particularité. Le reste de l’examen somatique a été normal. L’électrocardiogramme n’a pas montré de troubles de rythme ou de repolarisation. Un traitement par alpha-methyldopa à la dose de 750 mg/jour a été débuté. Devant la persistance des pics tensionnels sous traitement, avec des céphalées et des épisodes de sueurs, le diagnostic de phéochromocytome a été évoqué et la patiente a été mise sous prazosine à dose progressivement croissante: débuté à 0,5 mg 3 fois par jour augmenté par palier de 0,5 mg par prise tous les 3 jours avec une dose efficace à 10 mg sans épisodes d’hypotension artérielle. L’adrénaline plasmatique a été indétectable et la noradrénaline très élevée à 820 nmol/l (Normale < 580 nmol/l).

Le dosage des catécholamines urinaires des 24 heures a montré: métanéphrines à 78 mg (Normale = 40 à 150 mg); normétanéphrines à 1706 mg (Normale = 110 à 320 mg). Les bilans thyroïdiens et parathyroïdiens réalisés dans le cadre de la recherche de néoplasies endocriniennes multiples (NEM) ont été normaux. Une IRM abdominale a révélé une masse sus-rénale gauche de 8 cm de diamètre hétérogène avec une nécrose centrale sans autres localisations. Le bilan biologique standard a été sans particularité.

L’intervention chirurgicale (surrénalectomie gauche par voie trans-péritonéale) a été programmée à 14 semaines d’aménorrhée. Au bloc opératoire, la prise en charge anesthésique a été la suivante: prise de 2 voies veineuses périphériques de bon calibre; Préoxygenation de 5 minutes à FiO2:1; Monitorage: cardioscope, pression non invasive, oxymétrie de pouls, capnographie, température, sonde urinaire; antibioprophylaxie parentérale: 2g amoxicilline-acide clavulanique; induction anesthésique : 300 µg de fentanyl - 500 mg de thiopental -  6mg de vecuronium; prévention médicamenteuse de l’inhalation gastrique par 200 mg de cimetidine et 10 mg de metoclopramide; intubation facile par sonde n° 7; entretien de l’anesthésie par isoflurane et mélange 02/N20 à 50 %vol avec des réinjections de fentanyl; pose d’une ligne artérielle par voie radiale droite pour monitorage de la pression artérielle sanglante et d’une voie veineuse jugulaire interne droite pour surveillance de la pression veineuse centrale et perfusion éventuelle des catécholamines.

La manipulation de la tumeur a entraîné d’une part 4 épisodes d’hyperglycémie (entre 3,5 et 5 g/l) justifiant une surveillance toutes les 30 minutes de la glycémie capillaire et une insulinothérapie avec une dose totale de 40 unités; et d’autre part des pics d’hypertension artérielle (170-180 /110-120mmHg) jugulés par approfondissement de l’anesthésie (isoflurane MAC 3% avec des bolus de 50 mg de thiopental), renforcement de l’analgésie par des réinjections de fentanyl (dose totale de 600 µg) et une perfusion à la seringue électrique de nicardipine à 1 – 2 mg/h.

L’exérèse de la tumeur a été suivie par des épisodes d’hypotension artérielle (65 -70/45-50mmHg) jugulés par l’adrénaline en perfusion continue à une dose comprise entre 0,1 et 0,3 µg/kg/min.

La durée de l’intervention a été de 4 heures, la diurèse per opératoire à 1200 ml et le saignement estimé à 350cc.

L’extubation a été réalisée après réveil complet. L’analgésie postopératoire a été à base de proparacétamol et nefopam. La perfusion continue de l’adrénaline a été diminuée progressivement et arrêtée 12 heures après la fin d’intervention. Le traitement antihypertenseur a été repris devant la réapparition de pics hypertensifs. L’examen histologique a objectivé un phéochromocytome. L’examen obstétrical et l’échographie fœto-placentaire post opératoire n’ont pas montré d’anomalie. Au 7ème jour postopératoire, les métanéphrines urinaires se normalisèrent, et à 20 SA, la pression artérielle a été redevenue normale sans traitement. La patiente a accouché à 38 SA par voie basse après confrontation céphalo-pelvienne, d’une fille de 3.200 kg  dont l’examen néonatal a été normal. La mère et l’enfant ont quitté la maternité au 4^ème^ jour.

## Discussion

Le phéochromocytome est une tumeur à cellules chromaffines produisant des catécholamines en excès et située dans 90 % des cas dans la médullosurrénale. C’est une maladie rare qui peut être révélée par la grossesse. La rareté de cette association et sa similitude avec l’hypertension artérielle gravidique expliquent la fréquence des diagnostics méconnus pendant la grossesse. Plusieurs symptômes ont été rattachés au phéochromocytome, mais aucun d’eux n’est spécifique. Certains regroupements syndromiques, le caractère paroxystique des symptômes ainsi que leur association à l’hypertension artérielle sont évocateurs. La triade “céphalées/palpitations/sueurs” est retrouvée dans près de 90% des phéochromocytomes [2,3]. Les manifestations cardiovasculaires sont d’autant plus intenses que le taux préopératoire de catécholamines et de leurs métabolites est élevé [3,4]: HTA par stimulation des récepteurs 1adrénergiques du muscle lisse vasculaire; troubles du rythme par stimulation des récepteurs 1 et 1cardiaques; hypotension ou tachycardie isolée se voient plus fréquemment dans les phéochromocytomes à dopamine ou après une désensibilisation sympathique.

Lorsque le phéochromocytome est découvert tardivement, il peut induire une cardiomyopathie catécholaminergique qui rend plus difficile la prise en charge (mauvaise tolérance au remplissage vasculaire, aux poussées hypertensives ou aux produits inotropes négatifs, ischémie coronaire. . .) [5]

L’hyperglycémie due à une glycogénolyse induite par les catécholamines est fréquente et impose une surveillance péri opératoire étroite de la glycémie [2,5].

Le diagnostic doit également être évoqué devant une hypertension artérielle résistante aux traitements, même en l’absence de troubles vasomoteurs. Les autres symptômes sont moins évocateurs: douleur constrictive abdominothoracique ascendante, anxiété, tremblements, pâleur et troubles digestifs [3,6].

La recherche systématique d’un phéochromocytome chez la femme enceinte hypertendue n’est pas justifiée ; l’hypertension gravidique ou la pré éclampsie sont incomparablement plus fréquentes. Dans notre observation, Les principaux arguments évocateurs sont une uricémie normale, une protéinurie négative et l’existence de pics hypertensifs résistant aux traitements antihypertenseurs. La méconnaissance du diagnostic expose aux risques d’une décharge adrénergique qui peut être fatale pour la mère et le fœtus. Ces poussées sont spontanées ou déclenchées par une anesthésie inadaptée, une compression de la tumeur par l’utérus gravide, les contractions utérines, les efforts expulsifs ou les mouvements fœtaux [4,7].

La biologie permet d’affirmer le diagnostic par le dosage des catécholamines (adrénaline, noradrénaline, dopamine) sanguines ou urinaires, et de leurs métabolites urinaires (métanéphrine, normétanéphrine et acide vanylmandélique (VMA)) qui ne sont pas modifiées par la grossesse [5,6]. Le volume tumoral, estimé par l’imagerie, est le principal facteur d’influence de la durée de chirurgie, des pertes sanguines et de l’incidence des épisodes hypertensifs périopératoires [8]. L’échographie, dont la sensibilité atteint 89 à 97 % pour les localisations surrénaliennes en dehors de la grossesse, ne permet pas d’explorer le médiastin et est techniquement difficile en fin de grossesse avec un utérus gravide [6,9]. La tomodensitométrie est réalisable, mais l’IRM est l’examen de référence pendant la grossesse, car non irradiant et très sensible. La scintigraphie au MIBG (méta-iodobenzylguanidine) est contre-indiquée pendant la grossesse [3].

Le contrôle préopératoire de l’hypertension et des troubles du rythme contribue à la stabilisation hémodynamique per opératoire et à la diminution de la mortalité. Il repose classiquement sur les antagonistes α-adrénergiques. Les plus utilisé actuellement sont la prazosine ou la doxazosine qui évitent la tachycardie par un blocage spécifique des récepteurs α -1. Il est important de dιbuter ΰ faible dose et sous surveillance ιtroite de la pression artιrielle compte tenu du risque d’hypotension sévère /10-12/. Les inhibiteurs calciques comme la nicardipine sont de plus en plus utilisés. Ils présentent l’avantage de ne pas induire d’hypotension orthostatique et d’avoir moins d’effets secondaires que les α – bloquants [10,13]. Les β-bloquants ne sont pas indiqués de première intention et sont utilisés en cas de tachycardie ou arythmie sans dysfonction cardiaque. Le moment de l’intervention chirurgicale est très controversé. Avant 24 semaines d’aménorrhée, la chirurgie est possible. Depuis quelques années, l’abord chirurgical par cœlioscopie trans-péritonéale est de plus en plus souvent proposé. Il ne semble pas supérieure à la chirurgie à ventre ouvert dans la prévention des accès hypertensifs, car la création d’un pneumopéritoine stimule la libération de catécholamines par la tumeur de façon parallèle à la pression d’insufflation qui doit donc être maintenue aussi basse que possible ( inférieure à 12 mmHg ) [14,15]. En revanche, la coelio-chirurgie apporte un bénéfice net sur la douleur postopératoire, le délai de reprise du transit, la déambulation et la durée d’hospitalisation. Il est donc licite de proposer cette technique, à condition de la réserver à des opérateurs expérimentés. Après 24 SA, la laparoscopie est difficile en raison du volume utérin. L’idéal est de programmer une césarienne dès obtention de la maturité pulmonaire fœtale [9,12,13].

Le taux de mortalité maternelle lors d’une tumorectomie est de 31 % après accouchement par voie basse et de 19% après césarienne [10,14]. Cependant, lorsque le pronostic maternel est engagé, une intervention précoce est nécessaire quel que soit le terme. L’anesthésie proposée doit être profonde et très analgésique. L’induction peut faire appel au thiopental ou propofol et à un morphinique à forte dose. Les curares les plus histamino-libérateurs (atracurium, mivacurium) sont à éviter ainsi que la kétamine. L’entretien de l’anesthésie fait appel à des agents vasodilatateurs (halogénés ; tel le sevoflurane) afin d’abaisser les résistances systémiques lors des décharges de catécholamines et d’élimination rapide, afin d’alléger rapidement l’effet vasodilatateur en cas de collapsus [10,15].

La surveillance de la pression artérielle invasive et la pression veineuse centrale est indispensable pour pouvoir ajuster le remplissage vasculaire et le traitement antihypertenseur. Le Doppler œsophagien a l’avantage d’être non invasif, de fournir une surveillance continue du débit cardiaque et d’être rapidement mis en place, mais la sonde de Swan-Ganz fournit en plus des informations sur les pressions de remplissage et les résistances vasculaires systémiques. La mesure de la variabilité respiratoire de la pression artérielle (delta down) a été proposée pour renforcer le diagnostic d’hypovolémie efficace et semble prédictive de l’hypotension après clampage [11,16]. Le contrôle des accès hypertensifs par l’approfondissement de l’anesthésie/analgésie et/ou par un traitement antihypertenseur (urapidil, nicardipine) doit être une priorité péri opératoire. Il peut même être nécessaire de demander au chirurgien de cesser la manipulation tumorale le temps que celui-ci agisse. En cas de tachycardie sinusale, l’esmolol (Brevibloc), β-bloquant de courte durée d’action, est l’agent de choix (0,5 mg/kg administré sur une minute, suivi d’une perfusion à la vitesse de 150 μg/kg par minute).

L’hypotension due à la chute du taux des catécholamines après exclusion tumorale et aggravée par une hypovolémie efficace préopératoire en situation de vasoconstriction chronique, est corrigée par un remplissage guidé par les paramètres hémodynamiques. Le recours aux amines vasopressives pendant quelques heures est justifié lorsque le remplissage ne permet pas de normaliser la pression artérielle. La surveillance postopératoire doit être assurée en réanimation. L’analgésie doit être de qualité pour éviter les accès hypertensifs. Un traitement anti thrombotique à dose préventive est celui habituellement utilisé pour toute chirurgie abdominale. L’hypoglycémie est fréquente et justifie une surveillance répétée de la glycémie. En cas de surrénalectomie bilatérale, l’insuffisance surrénale postopératoire est obligatoire et doit être prévenue par un traitement substitutif approprié débuté le plus rapidement possible, à base d’hémisuccinate d’hydrocortisone (100 mg toutes les huit heures pendant 24 heures à j1, posologie à réduire ensuite progressivement pour atteindre en cinq jours environ une dose per os de 30 mg/j) et fludrocortisone (commencé à la dose de 50 μg deux fois par jour à j2 ou j3 de la décroissance de l’hémisuccinate d’hydrocortisone) [16,17] . Un diagnostic précoce au cours de la grossesse et une bonne prise en charge thérapeutique réduisent la mortalité fœtale entre 11 et 15 % et la mortalité maternelle entre 2 et 4 % [18,19].

## Conclusion

La survenue d’un phéochromocytome au cours de la grossesse pose un problème de diagnostic et de contrôle tensionnel. La prise en charge est pluridisciplinaire et diffère selon le terme. La mortalité a considérablement chuté dans les études les plus récentes grâce à une optimisation de la prise en charge.

## Conflits d’intérêts

Les auteurs ne déclarent aucun conflit d’intérêts
